# Inferring Cinematic Aesthetic Biases from the Statistics of Early Movies

**DOI:** 10.3390/e27070707

**Published:** 2025-06-30

**Authors:** Daniel M. Grzywacz, Norberto M. Grzywacz

**Affiliations:** 1 Yellow Dinosaur Productions, Chicago, IL 60601, USA; dmj.grzy@gmail.com; 2 Department of Psychology, Loyola University Chicago, Chicago, IL 60660, USA; 3 Department of Cognitive Science, Johns Hopkins University, Baltimore, MD 21218, USA

**Keywords:** normalized Shannon entropy, perceptual complexity, aesthetic values, optical flow, movies, statistical surprise, motion coherence

## Abstract

Cinematic aesthetic values have not been studied as thoroughly as those in music and the visual arts. Three hypotheses for these values are that they are like those of artistic paintings, that they emphasize the spatial coherence of the optical flow, and that they are temporally smooth. Here, we test these hypotheses and investigate other candidate aesthetic values by comparing the statistics of narrative movies and those obtained spontaneously. We perform these tests by using narrative movies from the early stages of cinematic history because these films are simple. We statistically compare these films with spontaneous movies of scenes from daily life. These statistical comparisons do not support the first hypothesis for early movies. The comparisons show that symmetry, balance, and image complexity (normalized Shannon entropy) are not different in early and spontaneous movies. For similar reasons, our data do not support the spatial coherence of early-movie optical flows as having cinematic aesthetic functions. However, in support of the third hypothesis, the temporal smoothness of luminance, but not of motions, appears to have cinematic aesthetic value. The data also uncovered two other cinematic aesthetic value candidates in both statistical surprise and spatial and temporal complexities. We discuss these candidates, pointing out similarities to music and the importance of film editing.

## 1. Introduction

Values are key to how the brain makes decisions [1,2,3]. When making decisions, one weighs the options with one’s unique values to choose the best alternative. An important set of values are those related to aesthetics. We use them when making decisions in fashion [4], technology [5], architecture [6], sexual selection [7], and many other domains. Given the importance of aesthetic values, they are partially encoded in our genes [8,9,10] and processed by the brain’s valuation system [11], which has networks dedicated to the computation of aesthetic values [12,13,14,15]. In this article, we use the terminology of aesthetic variables [6,16,17,18]. These are variables that one can measure in sensory stimuli and that can have an impact on an aesthetic decision. For example, the degree of symmetry of an image is one such aesthetic variable [19,20,21,22]. The larger the value that the symmetry variable reaches, the larger the probability that the image is beautiful. Thus, if one can measure an aesthetic variable in a sensory signal, then we say that the corresponding property has aesthetic value.

In this article, we probe the aesthetic values employed in narrative movies, that is, films that tell fictional stories. These values have not been studied as thoroughly and systematically as those in music [23,24,25,26,27] and the visual arts [19,20,21,22,28,29,30]. We thus call the aesthetic variables associated with narrative movies the “candidate cinematic aesthetic variables.” What cinematic variables are suitable to be these candidates? The place to look for answers to this question is where the foundational theories in visual perception and film studies meet.

We constrain the hypotheses in this article to early narrative movies. They are from the first period in the history of narrative cinema—that is, from 1903 to 1912 [31,32]. Our rationale for using this period is that its movies are the simplest. The cameras are static, and the development of the stories is theatrical [31,33,34,35,36]. The film stock is orthochromatic with low light sensitivity [37,38], leading to scenes with light or entirely white skies, increased atmospheric haze, accentuated texture, darker photography, and greater contrast. More than narrative, early films are about displaying magic and illusions to awe audiences [39]. Until 1907, filmmakers concerned themselves with the individual shot [31]. They set the camera far enough from the action to show the entire length of the human body as well as the spaces above the head and below the feet. Interventions through such devices as editing or lighting are infrequent. These devices become more sophisticated during the transitional period—that is, from 1907 to 1912 [32]. The increased use of editing and the decreased distance between camera and actors most obviously distinguish the films of the transitional period from their predecessors. In contrast, in 1913, a cinematic revolution began, making movies more sophisticated. That year saw the emergence of films like *Quo Vadis*, a two-hour epic that set a new standard for film length and production scale [40]. In addition, 1913 saw filmmakers like D.W. Griffith begin to experiment with large-scale stunts and real locations [41].

Because early movies are comprised of single shots with a series of frames [42,43,44], our first hypothesis is that the perceptual aesthetic variables of artistic paintings also apply to film. This hypothesis implies that each frame of a movie should strive to be symmetric and balanced [19,20,21,22,28,29,30], and to have the appropriate complexity [45,46,47,48,49,50] and other information-theoretic properties [51,52]. We also extend this hypothesis to pairs of consecutive frames, which define the optical flow [53,54,55]. The optical flow is a spatial map of velocity vectors obtained from two consecutive frames. Humans are sensitive to its metric components [56,57,58]. Because the optical flow is a spatial map, our first hypothesis postulates that the aesthetic properties of artistic paintings apply to the optical flow.

A second hypothesis applies only to the optical flow in consecutive pairs of movie frames, not individual ones. The origin of this hypothesis is the processing fluency theory [59]. It postulates that people experience more positive aesthetic responses when these individuals can process information about an object easily. This ease happens when a computation is important to the brain, which then dedicates resources to this computation. One of these computations is motion coherence [60,61,62,63,64], which the brain uses to take care of the problem of the measurement of optical flow being ill-posed [65,66,67]. Thus, our second hypothesis is that a candidate cinematic aesthetic variable is that the optical flow is spatially smooth.

Finally, we raise a third hypothesis for candidate cinematic aesthetic variables. This hypothesis is based on the perception of motion coherence occurring not only in space but also in time [68]. Hence, we hypothesize that a candidate cinematic aesthetic variable is temporal smoothness. This variable embodies the idea of fluid movement that is important in dance [69,70,71]. The temporal smoothness hypothesis can apply to both the luminance in individual frames and optical flow across time. The latter possibility shows that we may have two kinds of temporal domains for cinematic aesthetic variables. On the one hand, we may have short-term cinematic variables whose measurement is inside a single frame or a single optical flow map. On the other hand, we may have long-term cinematic aesthetic variables. We would measure them over long spans of time—that is, periods much longer than needed for measuring optical flows.

In this article, we compare the pre-1913 narrative movies with clips of spontaneous scenes to try to infer aesthetic values of the early filmmakers. We are not the first to use this kind of comparison as a tool [72]. The idea is based on art tapping into the aesthetic value centers of the brain [12,15,73,74,75,76]. Natural sensory stimuli are important for the brain [77,78,79,80,81], but the artist may exaggerate aesthetic properties beyond what people typically sense from these inputs [72]. One can thus discover such an exaggeration by comparing the statistics of art to those from spontaneous stimuli.

## 2. Methods

### 2.1. Movie Clips

In this study, we used forty-one 10 s single-continuous indoor clips from thirteen movies (Table 1) made early in the history of narrative cinema (1903–1912). These clips were obtained from YouTube (youtube.com—see URLs in the Supplementary Materials at https://osf.io/c2mrg—accessed on 2 May 2025) by using the application MacX YouTube Downloader https://www.macxdvd.com/free-youtube-video-downloader-mac/ (accessed on 21 February 2025). In selecting films for this study, we used Letterboxd.com as a reference. Using the popularity metric of the website, we found important films from our date range. Then, in selecting clips, we constrained the scenes to indoor shots, so the backgrounds were more uniform. We also excluded films that used animation techniques such as stop motion, focusing on live-action cinematography. We also made and used seventeen 10 s indoor clips from spontaneously shot movies. This number of spontaneous clips was decided by a power analysis [82]. To perform it, we used the statistics of the narrative movies. In this analysis, we fixed the power to 0.8, set the ratio between the number of movies to two or more, and asked to find an effect of 50% in temporal complexity. The spontaneous movies were scenes from daily life, like eating, walking, cleaning, and other such activities. To make the scenes spontaneous, we set the iPhone 14 Pro Max camera (Apple Inc., Cupertino, CA, USA) on a tripod facing roughly where the scene would take place. This prevented the filmaker posing the subjects ahead of time so as to avoid the aesthetic values of the cinematographer being part of the composition [72]. Thus, different from the narrative clips, the spontaneous examples did not have aesthetic intent.

Because the movies used 24 frames/s, we analyzed 240 frames for each of the 58 clips, amounting to 13,920 images. Considering the spatial resolution of the frames, we analyzed the intensity time courses of more than 40 billion pixels across our clips. All the clips can be found in the Supplementary Materials at https://osf.io/c2mrg (accessed on 2 May 2025).

### 2.2. Processing the Movie Clips Before Analysis

To process the movie clips for analysis, we began by making the spontaneous films black and white (grayscale). The early movie clips were already in this format. Next, we continued by rejecting a part of the pixels in the clips. Part of the pixels were cut because black margins were included in the movies. We also cut other pixels because occasionally, the camera moved at the beginning or end of a scene. This happened because early film cameras were operated by rotating a crank, which sometimes destabilized the recording device [83,84].

We then extracted the optical flows from each pair of consecutive frames. For this purpose, we used the optical flow algorithm of Farneback [55] after trying those of Horn and Schunck and Lucas and Kanade with less success [53,54]. (The latter produced more false positives via a procedure explained below.) We then marked five positions in each clip to check the temporal progress of luminance and optical flow. This check helped us ensure that the calculations were working correctly when a motion straddled one of these positions. However, two of the five positions were such that no motion straddled there. These two positions helped us understand the early movie variability in parts of the scene without motions. The causes of this variability were the crank motion of the cameras and the flicker of the intensity in early movies [85].

To deal with early movie variability, we computed speed outliers in the temporal trace of every pixel. The final analysis only used space and time points that passed the outlier test because they showed a motion going over them. We used the speed signals to compute the outliers because directions of motion were more variable. An outlier was a speed that was more than 4.25 scaled median absolute deviations (MAD) from the median [86]. The choice of 4.25 MADs is equivalent to 6 sigma in a normal distribution [87]. We chose to use the 6-sigma approach because it is rigorous and leads to only 3.4 defects per million opportunities. It is used across fields—mainly in manufacturing [88] but also in computer vision [89] and psychological research [90], two areas related to this article. As we will show in Section 3.3, this approach leads to ≈8% false positives, making our robust statistical analysis correct.

### 2.3. Candidate Cinematic Aesthetic Variables

We measured twenty candidate cinematic aesthetic variables in each movie clip (Table 2). These measurements included the variables described in the hypotheses of Section 1 and others that we considered interesting possibilities. The definitions of these variables have either appeared elsewhere [48,72] or in the next section.

Importantly, all measurements of skewness and kurtosis used robust statistical techniques to avoid biases from the false positives [92,93]. These techniques worked because as we show below, their rate was only about 8%.

Throughout this article, we use the term “Signal Complexity” when referring simultaneously to luminance and speed complexity. This refers to either the luminance or the speed signals in space and time. As seen in Table 2, we measure mean and standard deviation for luminance but not for speed. This is because the units of speed depend on image resolution, or image size coupled to viewing distance. However, we do measure the speed coefficient of variation because the ratio drops the units.

### 2.4. Temporal Smoothness, Roughness, and Complexity

The only two candidate cinematic aesthetic variables mentioned in Table 2 but not defined elsewhere are temporal smoothness and complexity. We start this section by addressing the mathematical definition of the latter.

As discussed elsewhere [48,49,72], we define complexity as normalized Shannon entropy, also known as relative entropy [94,95]. Let

$$\vec{s}\left(\vec{r},\vec{t}\right)=\left(s\left(r_{1},t_{1}\right),\ldots ,s\left(r_{1},t_{N_{t}}\right),s\left(r_{2},t_{1}\right),\ldots ,s\left(r_{i},t_{j}\right),\ldots ,s\left(r_{N_{r}},t_{N_{t}}\right)\right)$$

be an $N_{r}\times N_{t}$-dimensional vector of measurable signals (speed or luminance in our case). In this equation, $\vec{r}$ and $\vec{t}$ are spatial positions and times, respectively. Consequently, $\vec{s}\left(\vec{r},\vec{t}\right)$ is a mathematical representation of the movie clip. Let the set of possible values that $s\left(r_{i},t_{j}\right)$ can attain be $\left\{s_{1},\ldots ,s_{k},\ldots s_{N_{s}}\right\}$ (for example, in an 8-bit grayscale image, we have $N_{s}=256$). For the movie-clip Q and position $r_{i}$, let $s_{k}$ occur $M_{k}^{\left(Q,r_{i}\right)}$ times during the time course $\vec{s}\left(r_{i},\vec{t}\right)$. Then, we define the probability of $s_{k}$ in this time course as

$$P_{Q}^{\left(r_{i}\right)}\left(s_{k}\right)=\frac{M_{k}^{\left(Q,r_{i}\right)}}{\sum_{j=1}^{N_{s}}M_{k}^{\left(Q,r_{j}\right)}}\;,$$

From this equation, we define the temporal entropy for movie-clip *Q* and position $r_{i}$ as

(1) $$H_{T}\left(Q,r_{i}\right)=-\sum_{k=1}^{N_{s}}P_{Q}^{\left(r_{i}\right)}\left(s_{k}\right)\log_{2}\left(P_{Q}^{\left(r_{i}\right)}\left(s_{k}\right)\right)\;.$$

In practice, if $P_{Q}^{\left(r_{i}\right)}\left(s_{k}\right)=0$ for a k, then this term is not included in the sum, avoiding the singularity of the logarithm. This is possible because $\underset{x\to 0}{\lim}x\log x=0$.

To create temporal complexity out of this entropy, we divide it by its largest value, given any arbitrary movie. This largest entropy comes from images for which every position and time have a measurable value randomly picked from all signals. Thus, $P_{Q}^{\left(r_{i}\right)}\left(s_{k}\right)=1/N_{s}$. Substituting this result for $P_{Q}^{\left(r_{i}\right)}\left(s_{k}\right)$ in Equation (1), one obtains the maximal temporal entropy:

(2) $$H_{max,T}={log}_{2}\left(N_{s}\right)\;.$$

Dividing Equation (1) by Equation (2) gives the temporal complexity at position $r_{i}$ as the normalized Shannon entropy:

(3) $$C_{T}\left(Q,r_{i}\right)=\frac{H_{T}\left(Q,r_{i}\right)}{{log}_{2}\left(N_{s}\right)}\;.\;$$

Because the denominator is $H_{max,T}$, we have $0\leq C_{T}\left(Q,r_{i}\right)\leq 1$. We obtain 0 for single-signals movies (that is, the simplest ones) and 1 for movies whose measurable variables spread homogeneously through all values across space and time.

For any given movie-clip *Q*, the temporal complexity expressed in Equation (3) depends on $r_{i}$ and thus a spatial map. Such a map will appear in two figures in the article. In addition, the mean temporal complexity in the map will also be shown when comparing pairs of situations. This mean is

(4) $${\langle C_{T}\left(Q\right)\rangle }_{r_{i}}=\frac{1}{N_{r}}\sum_{i=1}^{N_{r}}C_{T}\left(Q,r_{i}\right)\;.\;$$

Alongside temporal complexity, this section aims to define temporal smoothness to test one of the hypotheses presented in Section 1. Smoothness is the property of the signal changing little between consecutive frames. The meaning of “little” is that this change should be smaller than the variability of the signal. In this article, we defined variability as the standard deviation. However, because the distribution of speeds is not normal, we used the median absolute deviation (MAD) [96] to estimate the standard deviation. We did so through a normal distribution transformation [86,97].

Using the notation in this section so far, we first measure the change between two consecutive frames:

$$\Delta_{Q}\left(\vec{s}\left(r_{i},t_{j}\right)\right)=\left|\vec{s}\left(r_{i},t_{j+1}\right)-\vec{s}\left(r_{i},t_{j}\right)\right|\;.\;$$

Then, we measure the standard deviation of the signal over time, $\sigma_{Q}\left(\vec{s}\left(r_{i},\vec{t}\right)\right)$. Finally, we define these two quantities to define temporal roughness for movie-clip *Q* and position $r_{i}$ as

(5) $$R_{T}\left(Q,r_{i}\right)=\frac{\Delta_{Q}\left(\vec{s}\left(r_{i},t_{j}\right)\right)}{\sigma_{Q}\left(\vec{s}\left(r_{i},\vec{t}\right)\right)}\;.$$

This equation provides a first definition of temporal smoothness because the latter rises as temporal roughness falls. Furthermore, temporal smoothness reaches its maximal value when the temporal roughness converges to zero.

Like for temporal complexity, temporal roughness depends on $r_{i}$ and thus a spatial map. Such a map will appear in a figure in this article for both luminance and speed. Moreover, like temporal complexity, the mean temporal roughness will be shown when comparing pairs of situations. This mean is

(6) $${\langle C_{T}\left(Q\right)\rangle }_{r_{i}}=\frac{1}{N_{r}}\sum_{i=1}^{N_{r}}R_{T}\left(Q,r_{i}\right)\;.\;$$

### 2.5. Statistical Analyses

We performed two kinds of statistical comparisons in this article: first, we compared the candidate cinematic aesthetic variables when applied to luminance versus speed; second, we compared these variables when measured in early versus spontaneous movies. All the comparisons used two-sided *t*-tests [97,98] with the Bonferroni correction [99] for the multiple-comparisons problem. We also assessed the effect sizes with the Cohen’s d statistic [100].

## 3. Results

### 3.1. Temporal Dynamics of Individual-Frame Aesthetic Variables in a Movie Clip

**Figure 1:**
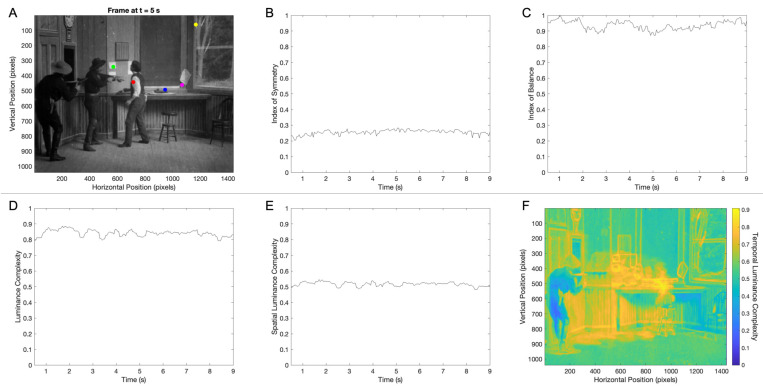
Aesthetic variables of the frames in a 9 s clip of the movie *The Great Train Robbery* (Table 1): (**A**) The Frame at 5 s into the clip. The colored dots show frame positions in which we are illustrating measurements later in the article. (**B**) The degree of symmetry of each Frame in the clip. (**C**) The degree of balance of each frame in the clip. (**D**) The luminance complexity (normalized entropy of the distribution of luminance) in each frame of the clip. (**E**) The spatial luminance complexity (normalized entropy of the two-dimensional distribution of luminance in pairs of pixels) in each frame of the clip (**F**). The temporal luminance complexity (normalized entropy of the distribution of luminance in each pixel across time—Equation (3)). The data in Panels (**B**–**E**) show that frame aesthetic variables are relatively constant over time.

A movie is a sequence of frames, each taken a brief amount of time after the last one. Therefore, the aesthetics of a movie may be divided into two parts: those of the frames and those of the changes between the frames. In this section, we focus on the former. We first measure the aesthetic variables in individual frames and then study how these variables vary over time. Figure 1 shows the results of this measurement for a 9 s clip of the classic movie The Great Train Robbery (Table 1).

A frame of The Great Train Robbery movie appears in Figure 1A. In it, one sees the moment in which two robbers are forcing the train-station manager to do their bidding in preparation for robbing a train. Before this frame, the door opens, and the robbers come in, forcing the manager to get up from the chair. After this frame, the manager does their bidding and goes back to his chair. We have measured the aesthetic variable values for each frame of this movie clip.

Figure 1B–E show that the aesthetic variables stay relatively constant across frames. The mean degrees of balance and symmetry across the frames of this clip were 0.26±0.06 and 0.936±0.002 (standard error), respectively. Thus, although the balance in the clip was consistent with that measured in Renaissance portraits (0.913±0.006), the symmetry was much lower than in the paintings (0.800±0.004) [18,72]. In turn, the mean luminance and spatial-luminance complexities in the clip were, respectively, 0.841±0.002 and 0.517±0.001. Hence, although the luminance complexity in this clip was slightly higher than that measured in Renaissance portraits (0.79±0.01), the spatial complexity was much lower than those paintings (0.70±0.01).

The temporal complexity aesthetic variable had no correspondence in paintings because they did not change in time. Nevertheless, this variable could help us understand aesthetic choices of movie makers. As seen in Figure 1F, the largest temporal complexity was in the region between the first robber and station manager where the action took place. The temporal complexity in this region was between 0.7 and 0.9. In contrast, the lowest temporal complexity (between 0.2 and 0.4) was in the region of the second robber, who almost did not move in the entire clip. Surprisingly, however, the temporal complexity in parts of the room with no motion (for example, in horizontal positions between 1200 and 1400) was not zero. This non-zero temporal complexity is due to technological issues in early cinema. They included a slight flicker and slight frame movement due to the mechanical action of the camera crank (Section 2.2).

### 3.2. Mitigating the Problem of False Positives in the Measurement Motion

**Figure 2:**
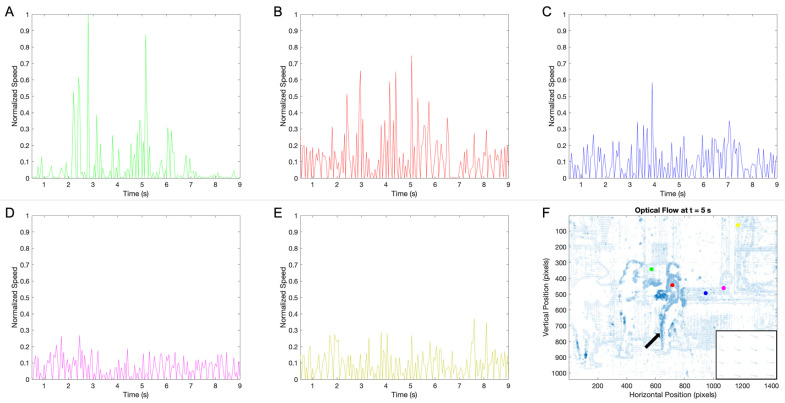
Normalized speeds at the positions shown by the colored dots in Figure 1A and the 5 s optical flow frame: (**A**–**E**) speeds at the marked positions, with the colors of the traces corresponding to the colors of the dots; (**F**) the optical flow at 5 s into the clip, with the right-bottom inset from the position shown by the arrow. The dots were chosen such that the green, red, and blue were crossed by motions during the clip. In turn, no motions traversed the magenta and yellow dots. Panels (**D**–**F**) show that even when a motion does not cross a position, one can measure motion because of the slight flicker and slight camera movement of the early movies (Section 2.2). Nevertheless, one can detect real motion crossings by spikes in the speed traces (Panels (**A**–**C**)). The inset shows the velocities when the motion of the station manager’s leg is towards the chair.

In Section 3.1, we focused on single-frame aesthetic variables and how they change over time. In this section, we begin to consider between-frame variables, namely, the optical flow vectors across space (Figure 2). To measure these vectors, we used the algorithm of Farneback [55] after trying those of Horn and Schunck and Lucas and Kanade with less success [53,54]. From these vectors, we extracted the speed and direction-of-motion components of the motion in each location of space. Figure 2 plots the speeds as a function of time for the five locations marked by the colored dots in Figure 1A.

The data in Figure 2 revealed that without further processing, a statistical analysis of between-frame aesthetic variables would be under the influence of false positives. Even in positions without motion (Figure 2D–F), a movement was detected because of the shortcomings of early movie cameras. However, the speed signals had a signature that showed the times and positions of motion crossing. These signals were spikes in the speed signals as a function of time (Figure 2A–C). These spikes were also plain as large vectors in the optical flows, marking contours of moving objects (Figure 2F).

**Figure 3:**
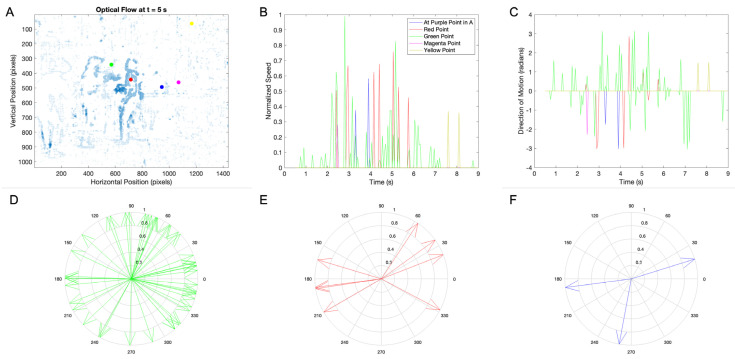
Optical flow and speed and direction-of-motion signals without outliers: (**A**) the optical flow at 5 s into the clip; (**B**) speeds at the marked positions, with the colors of the traces corresponding to the colors of the dots; (**C**) directions of motion (in radians) at the marked positions, with the colors of the traces corresponding to the colors of the dots; (**D**–**E**) distribution of directions of motion at the green (**D**), red (**E**), and blue (**F**) dots. The outlier-removal procedure cleaned the signals, as seen by comparing the optical flows in Panel (**A**) and Figure 2F. However, the cleaning was not complete, as shown by the residual motions in Panel (**A**). Although the cleaning improved the speed signals, those for the direction of motions were still sometimes complex (for example, Panel (**D**)).

To mitigate the problem of false positives, we took advantage of the spikes in the speed signals. We considered a motion to be real only when the measured speed was a statistical outlier (Section 2.2). These outliers were detected individually for each position because the background noise was different in separate places (compare, for example, Figure 2B,D). We thus eliminated from the analysis any data point that was not an outlier in time and space. One can see this cleaning of the data in Figure 3.

The outlier procedure did clean the signals, as seen in Figure 3. The strongest evidence of the cleaning was the lower number of non-zero velocity vectors in the areas of the clip without motions. To see this evidence, compare, for example, the horizontal positions between 1200 and 1400 in Figure 2F and Figure 3A. Further evidence of the cleaning came from the speed signals (Figure 3B). Only two of the spikes occurred in the positions of colored dots without motions (yellow in this case). Overall, the percentage of false positives in the positions without motion after cleaning was 13.5%±0.8%. Across all early movie clips in this study, the percentage of false positives was 8.4%±0.7%, as found by the same method.

Figure 3 also shows the observed directions of motion registered at the positions of the colored dots traversed by movement in this movie clip. First, Figure 3C shows the temporal progressions of the directions of motions. These progressions were, of course, consistent with what happened in the movie clip. For example, at the position of the red dot, we first saw negative directions around 2 to 4 s. They were the result of the station manager getting up from the chair and moving towards the robbers. Later, however, the directions of motion became positive, showing the return of the station manager towards the chairs. The distribution of these directions clarified further the movement tendencies (Figure 3D–F). For example, at the red dot, we saw two clusters of directions (around 30° and 190°), consistently with the motions of the station manager (Figure 3E). However, directions of motion were sometimes difficult to analyze, as illustrated by their distribution at the location of the green dot (Figure 3D). At this location, multiple directions of motion were elicited by the gun, hat, and face of the robber and the face and hand of the station manager. Consequently, we decided to focus on speed instead of on the directions of motion in the rest of this article.

### 3.3. Temporal Dynamics of Between-Frame Aesthetic Variables in a Movie Clip

**Figure 4:**
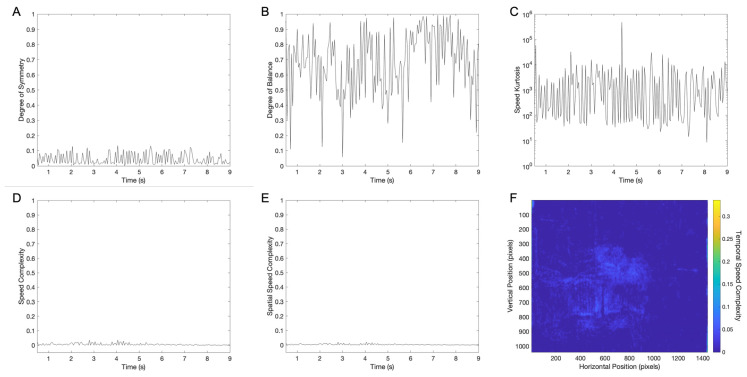
Candidate aesthetic variables of speeds in a 9 s clip of the movie The Great Train Robbery: (**A**,**B**,**D**–**F**) same conventions as in Figure 1B–E but for speeds instead of luminance; (**C**) kurtosis of the distribution of speeds. The results show that the degrees of symmetry and balance, and the various complexity measures are lower for speeds than for luminance (Figure 1B–E). In turn, the speed kurtosis is higher than observed for normal distributions.

Having set up the method for measuring between-frame aesthetic variables, that is, optical flow, we considered it at all times of the movie clip. The simplest hypothesis was that the candidate aesthetic variables for the optical flow were the same as those for individual frames (Section 1 and Figure 1). Thus, we considered the degrees of symmetry and balance and the complexities (normalized entropy) of the speed signal. Moreover, we considered its kurtosis. We did so because of the spike nature of the speed signal (Figure 3B) and thus the potential of the distribution of speeds to have long tails. The results of this analysis for the movie clip under consideration appear in Figure 4.

Different from luminance aesthetic values (Figure 1), this early movie clip had lower symmetry and balance (Figure 4A and B, respectively). The clip also had low complexity measures (Figure 4D–F). Nevertheless, except for balance, these measures were relatively constant over time, as their counterpart for luminance (Figure 1B–E). In contrast, the degree of balance varied widely over time, going from 0.1 to 1. In this clip, the mean degree of speed symmetry, the degree of speed balance, speed complexity, spatial speed complexity, and temporal speed complexity were, respectively, 0.044±0.003, 0.68±0.02, 0.0060±0.0006, 0.0034±0.0003, and 0.004936±0.000009. The low standard error of the temporal complexity was due to its independent measurements in nearly 1.5 million pixels (Figure 4F—Equation (3)).

The new candidate aesthetic variable in Figure 4 was the speed kurtosis (Section 1). As shown in Figure 4C, the speed kurtosis (measured robustly, Section 2.3) was high and varied over time. To acquire an idea of how high the speed kurtosis was, its mean value in Figure 4C is 6000±3000 (standard error). This value was much higher than the kurtosis for the normal distribution, namely, 3. Such high kurtosis suggested that pixels with motions were rare at any given moment, but when they happened, they caused the spikes seen in Figure 3B.

### 3.4. Temporal Smoothness and Temporal Roughness in a Movie Clip

**Figure 5:**
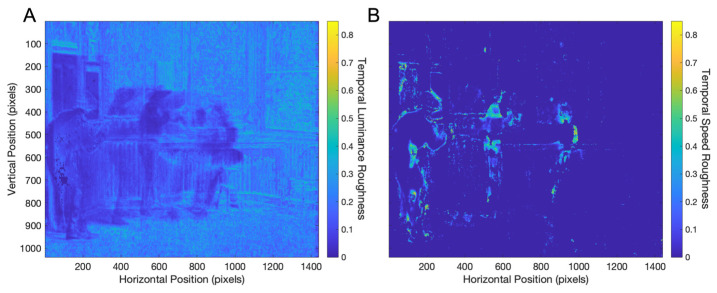
Temporal roughness (Equation (5)) in a 9 s clip of the movie The Great Train Robbery: (**A**) temporal roughness for luminance; (**B**) temporal roughness for speeds. The temporal roughness for speeds is lower than that for luminance, showing that motions change smoothly over time.

Another hypothesis for optical flow aesthetic variables was that one of them might be temporal smoothness (Section 1). For convenience of visualization, we calculated the opposite of smoothness, namely, roughness. Thus, the larger the smoothness was, the smaller the roughness was, and their relationship was linear. Temporal roughness captured something different from temporal complexity (Figure 1F and Figure 4F). Temporal complexity measured the normalized entropy of a signal over time—that is, how well distributed this indicator was over the entire trace. In contrast, temporal roughness captured how much the signal varied across consecutive time points. Figure 5 shows the temporal roughness of the movie clip under consideration.

Figure 5 shows that the temporal speed roughness is lower than that for luminance. The temporal luminance and speed roughness for this video clip were 0.18485±0.00007 and 0.01334±0.00007, respectively. Interestingly, the details of the spatial roughness maps illuminate certain other properties of early movies. For example, the temporal luminance roughness is larger for parts of the scene without motion than for those with it. The reason for the roughness outside motion is the early movie flicker (Section 1). The temporal luminance roughness was lower for parts with scenes with motion, showing that they were smooth. In contrast, the temporal speed roughness was larger for regions of scenes with motion because the speed is zero outside them. However, the temporal roughness was higher for speed than for luminance where motions occur.

### 3.5. Statistics of Aesthetic Variables Across Early Movie Clips

**Figure 6:**
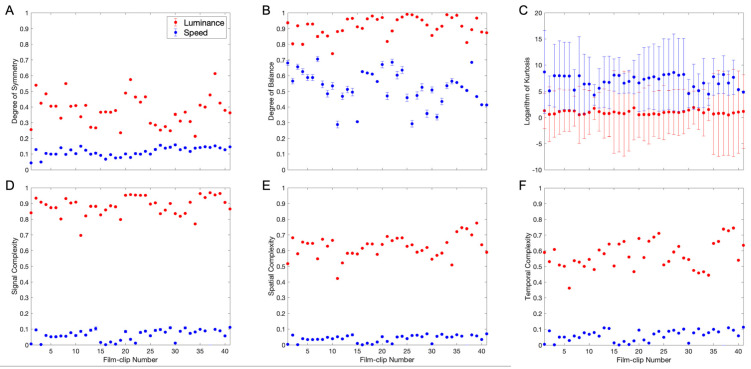
Comparison of the statistics of candidate aesthetic variables across forty-one clips of early movies: (**A**) mean degree of symmetry; (**B**) mean degree of balance; (**C**) logarithm of the mean kurtosis; (**D**) mean signal complexity; (**E**) mean spatial complexity; (**F**) mean temporal complexity (Equation (4)). The error bars are standard errors, and red and blue symbols mark luminance and speed signals, respectively. The results show that the findings with The Great Train Robbery clip (Film 1) generalize.

After exploring the methods and behaviors of the candidate optical flow aesthetic variables in detail, we continued to measure their statistics across different movies. We thus obtained results from forty-one clips extracted from thirteen early movies (Section 2.1). These results were for the same variables as illustrated in Figure 4. We compared them for luminance and speed to find out whether the differences seen in Figure 1 and Figure 4 generalize. Figure 6 shows the outcome of these comparisons.

The results in Figure 6 show that the conclusions reached with The Great Train Robbery movie clip generalize (compare to Figure 1 and Figure 4). The luminance signals were more symmetric (Figure 6A—paired-samples two-sided *t*-test t(40)=16.3, $p<6\times 10^{-19}$, Cohen’s d=3.68) and balanced (Figure 6B—t(40)=19.4, $p<2\times 10^{-21}$, d=4.29) than the speed signals, with large effect sizes. Furthermore, luminance signals showed more complexity (Figure 6D–F—luminance, t(40)=79.6, $p<9\times 10^{-45}$, d=16.8; spatial, t(40)=54.1, $p<3\times 10^{-38}$, d=11.4; temporal, t(40)=37.0, $p<6\times 10^{-32}$, d=7.60), with effect sizes also being large. The mean degrees of symmetry and balance for luminance were 0.38±0.02 and 0.91±0.01, respectively, while those for speed were 0.115±0.005 and 0.53±0.02. In turn, the mean signal, spatial, and temporal complexities for luminance were 0.88±0.01, 0.62±0.01, and 0.57±0.01, while those for speed were 0.10±0.06, 0.06±0.03, and 0.063±0.006.

A new result of Figure 6 concerned the luminance and speed kurtoses. We had already seen in Figure 4C that the speed was high for The Great Train Robbery clip. However, two questions remained: First, did the speed kurtosis continue to be high for other movie clips? Second, was the luminance kurtosis also high? The data in Figure 6C answered the first question in the positive and the second in the negative. The speed kurtosis was higher than the luminance kurtosis (Figure 6C—t(40)=−7.11, $p<2\times 10^{-8}$, d=−1.57), and the latter was statistically indistinguishable from the value of 3 expected by the Normal distribution. The mean luminance kurtosis was 2.9±0.2, while the mean speed kurtosis across all movie clips was 1800±200.

We concluded that aesthetic values associated with paintings and architecture [6,18,72] were less important in the optical flows of early movies and thus might not have cinematic aesthetic functions. However, these variables could still have such functions for individual frames. In contrast, that speed kurtosis was high for early movies suggested a role for surprise in cinematic aesthetics. Both kurtosis and surprise relate to the likelihood of extreme events (outliers) in a distribution. Kurtosis describes the “tailedness” of a distribution, showing how often extreme values occur. Surprise, in this context, refers to unexpected deviations from predicted or expected outcomes, often linked to outliers. Statistically, one can also measure Shannon surprise, a measure here related to complexity.

### 3.6. Comparing Luminance Aesthetic Variables in Early and Spontaneous Movie Clips

**Figure 7:**
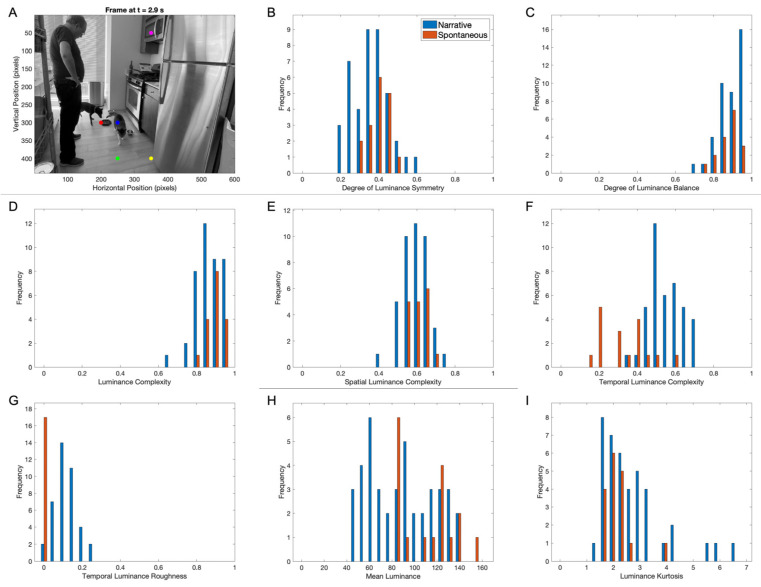
Comparison of histograms of mean luminance aesthetic variables in forty-one early and seventeen spontaneous movie clips. (**A**) The frame at 2.9 s of a spontaneous movie clip. (**B**–**I**) Histograms of candidate aesthetic variables as shown on the horizontal axes. The red and blue bins of the histograms mark early artistic and spontaneous movies, respectively. Two of the aesthetic variables in this figure are larger for early movies (temporal complexity and temporal roughness), and another is larger for spontaneous movies (mean luminance). However, most do not show a statistically significant difference.

An earlier study compared aesthetic variables in artistic paintings and spontaneously taken images to explore the painters’ values [72]. The logic was that painters would exaggerate compositional features that were aesthetically pleasing. And the study in question found exactly that for symmetry, balance, and complexity. We used the same logic of that study here to explore the cinematic aesthetic biases in early movies. The results of this exploration for luminance-related aesthetic variables appear in Figure 7.

The comparison of luminance aesthetic variables in early narrative movies and spontaneously filmed examples (Figure 7A, for example) revealed interesting differences and similarities. Five of the eight variables in Figure 7 were statistically indistinguishable in these two kinds of movies. The indistinguishable variables included the degrees of symmetry and balance, the luminance and the spatial complexities, and the kurtosis (Figure 7B–E,I). In contrast, the temporal complexity (Figure 7F—unpaired-samples two-sided *t*-test, t(56)=7.57, $p<5\times 10^{-10}$, d=2.16) and roughness (Figure 7G—t(56)=9.03, $p<2\times 10^{-12}$, d=2.58) were higher for early movies (Equations (4) and (6)), both with large effect sizes. However, the mean luminance was lower (Figure 7H—$t\left(56\right)=-2.88$, p<0.0057, d=−0.822), with the effect size also being large. The mean temporal complexity and roughness for early movies were 0.57±0.01 and 0.142±0.009, respectively, while the corresponding values for spontaneous examples were 0.35±0.03 and 0.017±0.003. In turn, the mean intensities for early and spontaneous movies were 92±5 and 114±5, respectively.

Therefore, early movies tended to be darker than current examples (for technological or artistic reasons—more on this in Section 4.2). More importantly, the temporal luminance properties of early movies were more complex, again suggesting the role of surprise in cinematic aesthetics (Section 3.5).

### 3.7. Comparing Speed Aesthetic Variables in Early and Spontaneous Movie Clips

**Figure 8:**
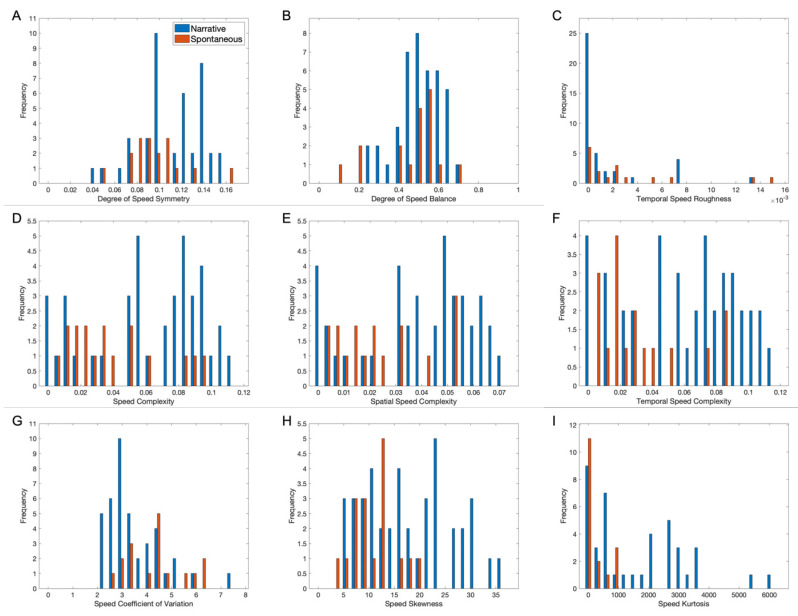
Comparison of histograms of mean speed aesthetic variables in forty-one early and seventeen spontaneous movie clips (**A**–**I**). Same conventions as Figure 7B–I.

Another question of interest was whether candidate speed aesthetic variables were different in early narrative movies versus spontaneously taken ones. The expectation based on Figure 1, Figure 4, Figure 5 and Figure 6 was that such differences existed. We expected to see more surprise in the speed signals of early movies than in spontaneous examples. This expectation might cause phenomena such as higher temporal complexity and roughness and higher kurtosis. We present a test of these expectations in Figure 8.

Figure 8 compares the statistics of nine candidate aesthetic variables measured in clips from the early and spontaneous movies. We found that early narrative movies yielded larger values in four of the variables of the earlier figures. These variables included kurtosis (Figure 8I—t(56)=3.63, $p<7\times 10^{-4}$, d=−0.822) and speed (Figure 8D—t(56)=2.34, p<0.025, d=−0.668), as well as spatial (Figure 8E—t(56)=2.54, p<0.015, d=−0.725) and temporal (Figure 8F—t(56)=3.01, p<0.004, d=−0.858) complexities (Equation (4)) variables, with the kurtosis and temporal complexity effect sizes being large, and the others being medium. In turn, none of the earlier variables yielded an advantage to early movies. Finally, three of the earlier variables did not yield a statistically significant difference, namely, the degrees of symmetry and balance and the temporal roughness (Figure 6). The mean kurtosis, speed, spatial, and temporal complexities for early movies were 1800±200, 0.064±0.005, 0.041±0.003, and 0.063±0.006, respectively, while the values for the spontaneous examples were 360±80, 0.042±0.007, 0.026±0.004, and 0.034±0.004.

Alongside these variables, Figure 8 includes two new variables—namely, the coefficient of variation and skewness (measured robustly, Section 2.3). The letter is related to the kurtosis because the speed signals are positive and have a long tail (large kurtosis). Thus, we expected a positive skewness. That is exactly what we found; that is, the mean speed skewness of the early movies (19±1) was statistically significantly larger (Figure 8H—t(56)=3.07, p<0.004, d=−0.875) than that of the spontaneous examples (12±1), with a large effect size. In contrast, the mean speed coefficient of variation for the earlier movies (3.6±0.2) was significantly smaller (Figure 8I—$t\left(56\right)=-2.75$, p<0.009) than that for the movies taken spontaneously (4.5±0.3). This is a consequence of so many speeds being zero in the optical flow.

In conclusion, the hypothesis of surprise in speed signals being a part of the aesthetic values of cinema values was supported by these data.

## 4. Discussion

### 4.1. A Study of Statistics Guiding Aesthetics Research

The premise of this article is different from the usual scientific approach. The article argues that if one studies the statistics of art, one may be able to infer the aesthetic values of the artist. We are not the first to make this argument [72]. The idea is based on art tapping aesthetic value centers of the brain [12,15,73,74,75,76]. Natural sensory stimuli are important for the brain [77,78,79,80,81], but the artist may exaggerate aesthetic properties beyond what people sense in these inputs [72]. One can thus discover such an exaggeration by comparing the statistics of art to those from natural stimuli [72]. In this article, we use this approach to try to capture aesthetic values driving early narrative movies—that is, those from 1903 to 1912. Our choice for these movies is motivated by their simplicity as pioneers—that is, their not involving methods that appeared later in the history of cinema. This is the same approach taken by the initial study of aesthetic values in historical artistic paintings [72].

In the rest of this Discussion section, we use the data in this article to consider the merits of various candidate cinematic aesthetic values (Section 4.3, Section 4.4, Section 4.5, Section 4.6 and Section 4.7). However, before embarking on this consideration, we address the limitations of the data.

### 4.2. Limitations

The easiest limitation to explain is that of the simplistic technology employed to shoot movies at the turn of the twentieth century. For example, the technology used cranks to move the film forward [83,84]. This movement caused instabilities in the position of the camera and the rate at which the film advanced. Another problem is the flicker of the intensity in early movies [85]. Combined with the movement of the camera, this flicker causes false-positive motions in the movie. We use an outlier-detection technique to mitigate the problem of false positives, but about 8% of them remain. Finally, we note that earlier movies were darker, on average, than current examples. Part of this darkness was due to older films having slower emulsions, which needed more light exposure [38]. However, we cannot easily distinguish this technological limitation from the artistic choice of making movies darker to set their mood [101,102].

Another limitation stems from brain processes affecting aesthetic values indirectly. We discuss two examples here: First, sensory adaptation [103,104] can also affect how people perceive the world and thus affect their aesthetic values. This limitation would occur, for example, if people staying in the same movie set for a long time were to change how they perceive it, thus changing their aesthetic choices. Second, people with neurological and psychiatric diseases and disorders may have distorted aesthetic values. In the simplest example, disorders of sensory systems alter how individuals process data about the world [105,106,107,108,109]. Visual artists, musicians, and filmmakers have been known to have such disorders [110,111,112,113,114,115] or related neurological disorders [116]. Certifiable examples of such disorders affecting aesthetic compositions are those of Claude Monet and Edgar Degas [110,111,115]. Monet suffered from cataracts and Degas from a progressive retinal disease. Thus, Monet’s late canvases (before surgery [114,115]) showed strange uses of color. Moreover, Degas’ late works were coarse, a major departure from his earlier finer works [117]. Other examples include colorblind artists who limit their palette to ambers and blues and avoid greens [111]. These changes would suggest a change in these artists aesthetic values, which might not be true. However, people rarely discuss having a neurological disorder; thus, we cannot be certain as to whether our data exhibit such an effect.

The final limitation is the lack of experimentation with candidate movie aesthetic values. Our approach from statistics to aesthetic values must only be taken as initial evidence and as a guide to future experiments. Performing perceptual experiments with a sufficiently substantial number of moviemakers may be difficult. However, our results are not just about the aesthetic values of moviemakers but should also apply to the general population. We are thus preparing experiments to probe preferences for candidate cinematic aesthetic variables and to test whether the findings in this article apply to most people.

### 4.3. Are Painting Aesthetic Values Applicable to Movies?

We now turn our attention to candidate movie aesthetic values. The first hypothesis raised in this article is that the aesthetic values related to artistic painting apply to movies (Section 1). Thus, values related to symmetry, balance, complexity, and other information-theoretic properties should affect films [19,20,21,22,28,29,30,51,52]. These values could apply to both individual frames of the movies and to their optical flows, which also form a spatial map. However, our data argue against the artistic-painting hypothesis for early-movie optical flows because their degrees of symmetry and balance, as well as their complexities, are too low. Furthermore, our data do not support the hypothesis for individual frames because these aesthetics variables are like the corresponding examples in spontaneous movies.

Why do early-movie cinematic aesthetics not consider these traditional artistic values? We hypothesize that they may be competing with other values that we will discuss in Section 4.4, Section 4.5, Section 4.6 and Section 4.7. Competition between aesthetic values has been considered elsewhere to explain why certain aesthetic values are not emphasized as much in art and architecture [6,17,18,72]. For example, if the aesthetic variables of symmetry and balance are too high, they will reduce the complexity of an image. What is it about movies that might prevent them from keeping values like these a high priority? We propose that if motion causes various parts of the frame to move, then maintaining painting-related aesthetic variables may be too difficult. Nevertheless, it would be an exaggeration to say that painting aesthetic variables are never applicable to narrative movies. Newer movies than those studied here contradict this notion [118,119,120]. Why do we not find evidence of such aesthetics in early movies? The limitations of the available technology may have led filmmakers to focus on aesthetic values other than those of individual shots.

### 4.4. Is Optical Flow Spatial Smoothness a Cinematic Aesthetic Variable?

Another candidate aesthetic variable for movies is the spatial smoothness of the optical flow (Section 1). Motion coherence is important for human vision [60,61,62,63,64] to address the problem that the measurement of local velocity is ill-posed [65,66,67]. However, our data are not favorable to the hypothesis that optical flow spatial smoothness is an aesthetic variable of early film. They reveal no statistical differences between early and spontaneous optical flows. Interestingly, however, early-movie speed smoothness is greater than that of luminance. This is because early movies tend to use stationary cameras, with only a few objects moving in the scene.

### 4.5. Is Temporal Smoothness a Cinematic Aesthetic Variable?

Related to spatial smoothness is its temporal counterpart. As in the spatial domain (Section 4.4), temporal coherence has been found to be important for the perception of visual motion [68]; people find motion fluidity aesthetically pleasing [69,121,122]. One can have two types of motion fluidity, one related to changes of luminance and another connected to the evolution of optical flows. These types are different from the optical flow because their temporal evolutions are long-range, i.e., they do not simply extend over a couple of consecutive frames (Section 1). Our data argue against the hypothesis that the temporal smoothness of early-movie optical flow is an aesthetic variable because it is the same as what one find with spontaneous motions. However, unexpectedly, the data support the alternative hypothesis that the temporal smoothness of luminance is an aesthetic variable. We propose that fluidity may have more to do with slow changes of luminance than slow changes of motion.

### 4.6. Is Either Temporal or Spatial Complexity a Cinematic Aesthetic Variable?

Other unexpected possible cinematic aesthetic variables appearing from the data relate to complexity (normalized Shannon entropy). Let us first consider these variables in the spatial domain. This time, we found no effect for luminance, but the spatial complexity of speeds was higher in the early movies than in the spontaneous examples. Now, turning our attention to temporal complexity, the data support the contention that it as a cinematic aesthetic variable for both luminance and speed. In both cases, the temporal complexities are larger in early movies than in spontaneous examples. Spatial complexity is valuable because it means richer speed signals in neighborhoods of space [123,124,125]. Similarly, temporal complexity means richer signals over time. In addition, spatial and temporal complexities are important in another way, making them potentially compatible with the processing fluency theory [59]. As explained in the introduction, this theory postulates that computations have aesthetic value if they are important to the brain, which then dedicates resources to their computation. In the case of spatial and temporal complexities, they are important because the brain uses these kinds of information to allocate resources for processing the incoming sensory signal [48,49,50].

### 4.7. Is Surprise a Cinematic Aesthetic Variable?

The final candidate aesthetic variable appearing unexpectedly in the movie data is that of surprise. The evidence for this variable comes from kurtosis, which detects values highly discrepant from the norm. Again, we see no evidence of luminance surprise, as luminance kurtosis is similar in early and spontaneous movies. However, the speed kurtosis is larger in early movies, with the evidence showing this to be true regardless of whether we measure kurtosis in time or space. This kurtosis result supports the hypothesis that surprise is a cinematic aesthetic variable. Temporal surprise may be aesthetically pleasing because it creates drama and tension [126,127,128]. Moreover, both spatial and temporal surprise create focal points, attracting the attention of the viewer [129,130,131].

Surprise as a cinematic aesthetic variable has two interesting implications: First, the aesthetics of movies may have a closer relation to those of music than those of visual art [132]. Although films can have beautiful scenes [133,134,135], they were not the emphasis in early movies. A big part of the emphasis is on surprise, as in music [23,24,25,26,27]. For example, the probability that a song will reach the top quartile of the *Billboard Hot 100* charts has a relationship with = average harmonic surprise (as measured as Shannon Entropy) [26]. This effect of surprise on musical preference has been corroborated in behavioral experiments [27]. The connection between film and music in terms of surprise may have something to do with their temporal aspects. Nevertheless, the surprise–beauty balance shifts towards the latter in newer movies (Section 4.3). Second, the most used techniques to insert surprise (high kurtosis) into movies are abrupt editing and the entrance and exit of people, animals, and objects [136,137]. We see exits and, especially, entrances in our early movies. However, early movies have limited (but not zero) editing, in part due to technological reasons. Another contribution to this limitation comes from early films still using the concepts of stage theater [31,35,36]. The ideas that came later in the history of cinema were, of course, unknown to early filmmakers. Thus, given the importance of surprise, modern films spend significant energy on editing, i.e., cutting from a scene to another and back [138]. Surprise via editing is, in a way, an aesthetic technique unique to films. We finish this discussion by letting the movie director Martin Scorsese forcefully explain this point in his own words [139] “I think that Stanley Kubrick said that … because [film] combines all the other arts … the only thing that’s originally film is editing … I always get amazed when I’m in the cutting room … I still get a thrill when you cut one shot next to the other and there’s a … movement that is conjured up in your head by the cut. It’s like a spiritual move …”

## 5. Conclusions

We conclude that the most important aesthetic variables of narrative movies may be different from those of visual art. The symmetry, balance, and complexity of individual frames may play a role but are not necessarily the most important aesthetic elements to filmmakers. Instead, these elements may include the smoothness and complexity of luminance temporal change. Other such elements are surprise in the optical flow, along with spatial and temporal complexity. The importance of surprise makes movies, aesthetically, like music.

## Figures and Tables

**Table 1:** Early movies used in this study. The title “# Clips” refers to the number of clips used from each movie.

Movie	Director	Year	# Clips
*The Great Train Robbery*	Edwin Stanton Porter	1903	1
*Jupiter’s Thunderballs*	Georges Méliès	1903	2
*Le Diable Noir*	Georges Méliès	1905	5
*Rescued by Rover*	Cecil Milton Hepworth	1905	1
*Les Résultats du Féminisme*	Alice Guy	1906	5
*L’Assassinat du Duc de Guise*	André Calmettes	1908	5
*The Electric Hotel*	Víctor Aurelio Chomón y Ruiz	1908	1
*La Battaglia del Grano*	D. W. Griffith	1909	4
*The Invisible Thief*	Ferdinand Zecca	1909	3
*The Panicky Picnic*	Camille de Morlhon	1909	2
*Frankenstein*	J. Searle Dawley	1910	4
*L’Inferno*	Francesco Bertolini, Adolfo Padovan, Giuseppe De Liguoro	1911	5
*Falling Leaves*	Alice Guy Blaché	1912	3

**Table 2:** Candidate cinematic aesthetic variables.

Name	Definition
Degree of Luminance Symmetry	Index of symmetry in Reference [72]
Degree of Speed Symmetry	Same as last row but for optical flow speeds
Degree of Luminance Balance	Index of balance in Reference [72]
Degree of Speed Balance	Same as last row but for optical flow speeds
Luminance Complexity	Complexity of Order 1 in References [48,72]
Speed Complexity	Same as last row but for optical flow speeds
Luminance Spatial Complexity	Complexity of Order 2 in References [48,72]
Speed Spatial Complexity	Same as last row but for optical flow speeds
Luminance Temporal Complexity	Equations (3) and (4) applied to luminance
Speed Temporal Complexity	Equations (3) and (4) applied to speed
Luminance Temporal Roughness	Equations (5) and (6) applied to luminance
Speed Temporal Roughness	Equations (5) and (6) applied to luminance
Mean Luminance	Mean across positions and times
Luminance Standard Deviation	Time average of spatial standard deviation
Luminance Coefficient of Variation	Ratio of last two rows
Speed Coefficient of Variation	Same as last row but for optical flow speeds
Luminance Skewness	Time average of spatial skewness
Speed Skewness	Same as last row but for optical flow speeds
Luminance Kurtosis	Time average of spatial kurtosis [91]
Speed Kurtosis	Same as last row but for optical flow speeds

## Data Availability

All data are in the Supplementary Materials reported above.

## Supplementary Materials

The following supporting information can be downloaded at https://osf.io/c2mrg: Movies data and computer code.
